# PlantTFcat: an online plant transcription factor and transcriptional regulator categorization and analysis tool

**DOI:** 10.1186/1471-2105-14-321

**Published:** 2013-11-12

**Authors:** Xinbin Dai, Senjuti Sinharoy, Michael Udvardi, Patrick Xuechun Zhao

**Affiliations:** 1 Plant Biology Division, The Samuel Roberts Noble Foundation, 2510 Sam Noble Parkway 73401 Ardmore, OK, USA

**Keywords:** Transcription factor, Transcriptional regulator, Chromatin regulator, High-throughput prediction, Plant genome

## Abstract

**Background:**

Plants regulate intrinsic gene expression through transcription factors (TFs), transcriptional regulators (TRs), chromatin regulators (CRs), and the basal transcription machinery. An understanding of plant gene regulatory mechanisms at a systems level requires the identification of these regulatory elements on a genomic scale.

**Results:**

Here, we present PlantTFcat, a high-performance web-based analysis tool that is designed to identify and categorize plant TF/TR/CR genes from genome-scale protein and nucleic acid sequences by systematically analyzing InterProScan domain patterns in protein sequences. The comprehensive prediction logics that are included in PlantTFcat are based on relationships between gene families and conserved domains from 108 published plant TF/TR/CR families. These prediction logics effectively distinguish TF/TR/CR families with common conserved domains. Our systematic performance evaluations indicate that PlantTFcat annotates known TF/TR/CR families with high coverage and sensitivity.

**Conclusions:**

PlantTFcat provides an analysis tool to identify and categorize plant TF/TR/CR genes on a genomic scale. PlantTFcat is freely available to the public at http://plantgrn.noble.org/PlantTFcat/.

## Background

Plants regulate intrinsic gene expression through transcription factors (TFs), transcriptional regulators (TRs), chromatin regulators (CRs) and the basal transcription machinery to control development and respond to environmental changes. To understand the fundamental mechanisms of plant development and environmental responses, we need a systematic way to identify and categorize TFs, TRs, and CRs on a genomic scale.

TFs are proteins that bind to specific DNA sequences, usually to a motif in the target gene promoter, to control the transcription of the target gene. TFs may perform this function with other proteins, *e.g.*, TRs. A classic example of the control of gene expression via TFs and TRs is the control that the auxin signal has over all major aspects of plant development. Auxin enhances the proteolysis of auxin/IAA (Aux/IAA) transcriptional repressors, which heterodimerize with the auxin response factor (ARF) TFs to block the transcriptional activation of auxin-responsive genes [1]. This example demonstrates that TFs and TRs play equally vital roles in plants.

CRs are also essential components in the gene expression regulation machinery. CRs are divided into two categories: 1) chromatin remodelers that reposition and restructure nucleosomes and 2) chromatin modifiers that add or remove covalent marks from histones. Chromatin remodeling complexes are widely conserved among plants, animals, and fungi. The maintenance of the chromatin structure conserves epigenetic marks on the histones, which are required for both proper plant development and the maintenance of perception to environmental cues such as light and temperature [2]. In *Arabidopsis thaliana*, mutations of histone mono-ubiquitination enzymes lead to premature flowering as well as defects in plant size and leaf development. These changes indicate that histone mono-ubiquitination enzymes are master regulators of plant development [3].

Over the past several years, next-generation sequencing (NGS) technologies have enabled life scientists to collect genome sequences both rapidly and economically. However, although plant gene transcription regulatory elements, including TFs, TRs, and CRs, have been broadly studied for several years, only a few tools have been developed to systematically categorize and analyze published TF/TR/CR gene families on a genomic scale. One popular database, DBD (http://www.transcriptionfactor.org), features PFAM [4] and superfamily [5] domain information and is organized by families and genomes [6]. Furthermore, DBD provides a supplemental function that allows users to submit single protein sequence to search for DNA binding domains within the protein. This database, however, focuses on TFs only.

Another popular database, PlnTFDB (http://plntfdb.bio.uni-potsdam.de/), hosts sequences and PFAM domain models of 84 TF/TR families in plants [7]. However, PlnTFDB lacks an analytic function and serves as a reference database only. ITAK is an unpublished analysis tool (http://bioinfo.bti.cornell.edu/cgi-bin/itak/index.cgi) that has adopted the PFAM domain rules described in PlnTFDB to predict TF and TR genes. However, although the web version of ITAK is user-friendly, this tool can analyze a maximum of 50 sequences for each submission, which makes it impractical for analyzing sequences on a genomic scale. The standalone version of ITAK may be capable of large-scale data analyses if the Linux command line program is optimized and deployed on a parallel computing cluster. However, this implementation poses a challenge for most biologists.

The above analysis tools were developed on the basis of domain patterns from PFAM and superfamily databases, which are not sufficient for the systematic identification and categorization of TF/TR domains because some TF/TR domains are not covered by either the PFAM or the superfamily databases. For example, the BTB/POZ-like domain is the featured domain of the BTB-POZ, BTB-POZ-MATH, and ABTB families, but the domain model is available only in the PROSITE profile database [8] (*i.e.* PS50097) or the SMART [9] database (*i.e.* SM00225). As another example, the Chromo and shadow domains of the CHROMO-DOMAIN family are also found only in the PROSITE and SMART domain pattern databases.

InterProScan [10] is a comprehensive program that integrates 14 domain signature search programs and corresponding domain pattern libraries and has been used for TF/TR gene prediction. Kakar *et al* (2008) scanned all *Medicago truncatula* genome sequences using InterProScan and identified TF/TR gene families from a curated mapping table between InterProScan domains and TF/TR families [11]. Wang *et al* (2010) also developed a TF/TR prediction protocol using InterProScan and the mapping table previously described in their soybean database, SoyDB [12]. However, to date, neither of these efforts has led to a web server or software that is available for public use.

We have developed a web-based analysis server, PlantTFcat (http://plantgrn.noble.org/PlantTFcat/), which utilizes InterProScan to systematically search proteins for TF/TR/CR-related domain signatures. Furthermore, we have developed comprehensive prediction logics based on relationships between gene families and conserved domains to effectively distinguish families with common conserved domains, which are often overlooked in traditional BLAST-based searches. PlantTFcat therefore systematically predicts and categorizes plant TF, TR, and CR genes with high coverage and sensitivity.

To provide high-throughput genome-scale analysis capability, we customized InterProScan to include only relevant domain information. We further accelerated the back-end prediction module by deploying PlantTFcat on our in-house BioGrid parallel computing platform, which is equipped with approximately 300 AMD processor cores at 2.5GHz clock frequency. With these optimizations, PlantTFcat is able to analyze the *Arabidopsis thaliana* genome release TAIR10 (http://www.arabidopsis.org/) in less than 10 minutes.

## Implementation

### Comprehensive prediction logics

The InterProScan domain patterns of 108 TF/TR/CR families were compiled in a back-end database by mining the published datasets [7,11] and reviewing the existing literature. For each family, the prediction logic is composed of a “*must-have domain”* pattern and a “*prohibited domain”* pattern, respectively. The domain signatures of a protein in InterProScan that meet the former pattern may satisfy the criterion for such a family. However, any signature that meets the latter pattern will lead to a negative prediction.

The “*must-have domain”* pattern was designed to handle logical relationships such as “AND” and “OR”. For example, the ARF TF family is expected to include either IPR010525 or IPR011525 (“OR”) as well as IPR003340 (“AND”). We therefore use a logical expression (IPR010525|IPR011525)&IPR003340 to represent the “*must-have domain”* pattern for the ARF TF family.

The “*prohibited domain*” pattern was designed to divide superfamilies into more specific families based on the existing literature. For example, experimental evidence implicates the BTB/POZ-like domain, which is a protein-protein interaction module, in the regulation of gene expression through the local control of chromatin conformation. In some cases, the BTB-POZ domain has been observed with MATH (meprin and TRAF-C homology), which is known to be involved in the regulation of protein processing and ubiquitination. However, the BTB-POZ domain has also been associated with the ankyrin repeat, which is another protein-protein interaction domain [13,14]. Therefore, we categorized BTB-POZ proteins into the BTB-POZ-MATH and ABTB families based on the differences in their companion domain using the “*prohibited domain*” pattern. As another example, TFs with the DNA-binding domain MADS box (IPR002100) can be subdivided into two families: MADS type1 and MADS-MICK. The MADS type1 family includes only the MADS box. However, the MADS-MICK family includes both the MADS box and a keratin-like box (K-box) (IPR002487) that promotes protein dimerization. To discriminate between these two families, the K-box domain can be included in the “*prohibited domain”* pattern of the MADS type1 family.

The comprehensive prediction logics, including the “*must-have domain”* and “*prohibited domain*” patterns, are available in Additional file 1 for all 108 families. These prediction logics are also listed by family on the PlantTFcat website (http://plantgrn.noble.org/PlantTFcat/familylist.do).

#### Infrastructure and implementation

The PlantTFcat consists of an intuitive web interface that allows users to submit large numbers of sequences and retrieve analysis results as well as a sophisticated back-end high-performance prediction module that uses InterProScan to search potential domains from the user-submitted sequences. This back-end module screens potential TFs/TRs/CRs by referring to the featured conserved domain patterns of each family. The PlantTFcat web server runs on an Oracle Java Virtual Machine and Resin J2EE server. The high-performance prediction module is deployed on our in-house BioGrid parallel computing platform, which runs on a CentOS 6 Linux Operating System. The *PlantTFcat* web interface was written in the Groovy language and the back-end pipeline was written in both Java and Groovy.

#### High-throughput capability

InterProScan is a computationally intensive program that completes the search for the domains of a typical protein sequence in approximately 5 minutes. To analyze genome-scale sequences, we reduced the domain pattern libraries in InterProScan to include only TF/TR/CR-related domains, which accelerated the search by 2 to 3 orders of magnitude. This customized InterProScan installation coupled with our in-house BioGrid parallel computing deployment allows PlantTFcat to analyze the entire *Arabidopsis thaliana* genome (~30,000 genes) within 10 minutes.

### Session management

User-submitted jobs are scheduled into the back-end pipeline by a queue management system on a first-come first-serve basis. Only two sessions can run simultaneously in the back-end pipeline. All other sessions wait in the queue until the current jobs have been completed. An exception is made for small jobs (<20 kb), which are submitted and run directly without waiting in the queue.

## Results

### Interface

**Figure 1:**
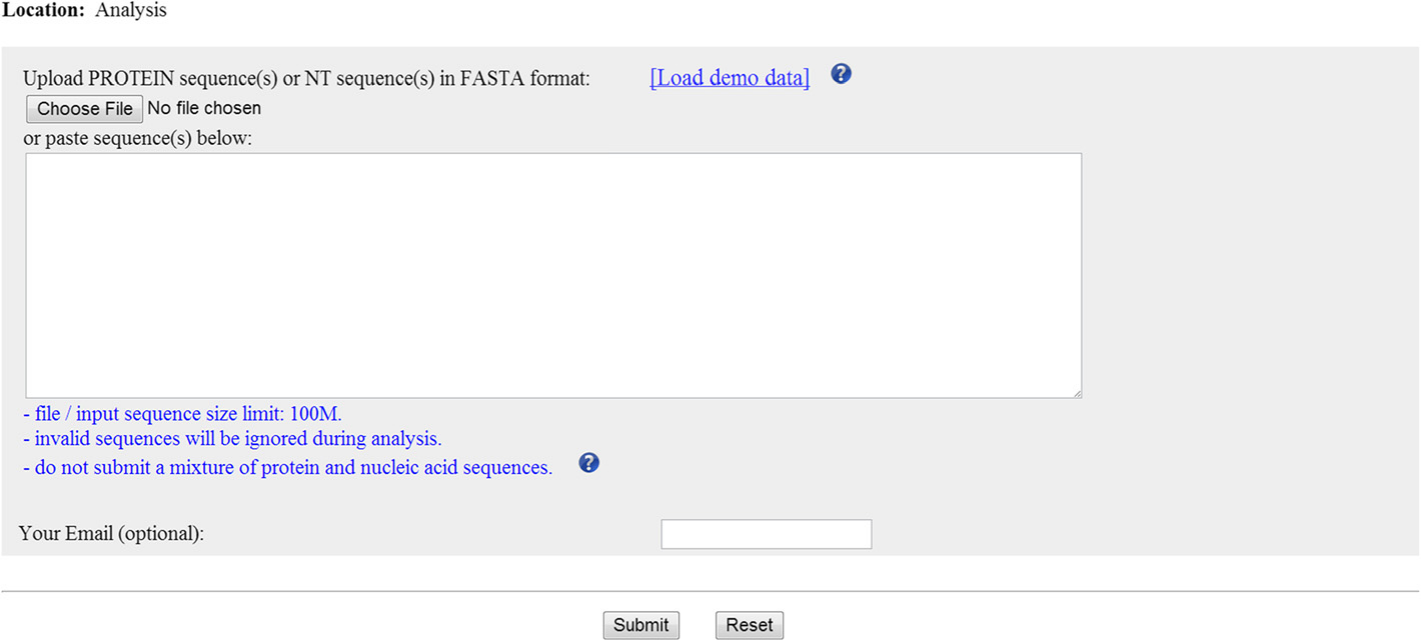
The data submission interface located under the “Analysis” tab of the PlantTFcat web server.

**Figure 2:**
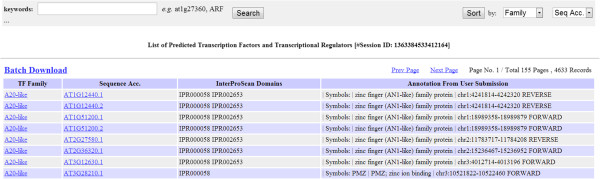
An example output page of PlantTFcat that includes search, sort, and batch download links.

Users can submit either nucleic acid or protein sequences under the “*Analysis*” tab (Figure 1) of PlantTFcat web server. The back-end pipeline detects the sequence type and translates nucleic acid sequences into protein sequences according to their six open reading frames (ORFs). Most submissions can be completed within several minutes. Large datasets, *e.g.*, approximately 100 MB of nucleic acid sequences, may require up to an hour to complete. PlantTFcat returns a web-based table that allows users to query, sort, and filter the results when the analysis session is complete. The prediction results are also available for batch download (Figure 2).

### Performance evaluation

We compared the predictions from PlantTFcat with the benchmark data in PlnTFDB [7] using the *Arabidopsis thaliana* genome release V8 (TAIR8) and the *Zea mays* genome release 3b.50 (http://www.maizesequence.org/). PlantTFcat missed 199 genes out of 2,757 TF/TR gene models from the *Arabidopsis thaliana* data in PlnTFDB. Moreover, PlantTFcat reported 1,744 additional gene models as TF/TR/CR candidates. Of these identified gene models, only 95 were not confirmed, because they were annotated as either unknown or non-TF/TR genes per the TAIR8 annotation (see Additional file 2). In the monocot species, *Zea mays*, PlantTFcat achieves a similar prediction performance: 370 genes were missed and 6,050 genes were newly predicted as TF/TR/CR genes (see Additional file 3) (Table 1). Such results suggest that PlantTFcat can predict TF/TR/CR genes with high coverage and sensitivity.

**Table 1:** Benchmark evaluation of PlantTFcat using the PlnTFDB reference database

	**Matches**	**Conflicts**	**Missed genes**	**Newly predicted genes**
*Arabidopsis thaliana*				
(32,825 gene models)	2,225	333	199	1,744
*Zea Mays*				
(147,837 gene models)	4,094	782	370	6,050

The ‘Matches’ column denotes the number of genes that were categorized into the same/synonymous/super/sub families of PlnTFDB by PlantTFcat. The ‘Conflicts’ column denotes the number of genes that were identified by PlantTFcat as TF/TR/CR genes, but with different family annotations than PlnTFDB. The ‘Missed genes’ column represents the genes that were omitted by PlantTFcat but identified as TF/TR genes in the PlnTFDB database. The ‘Newly predicted genes’ column denotes the genes predicted as TF/TR/CR genes by PlantTFcat that were missed by PlnTFDB.

**Figure 3:**
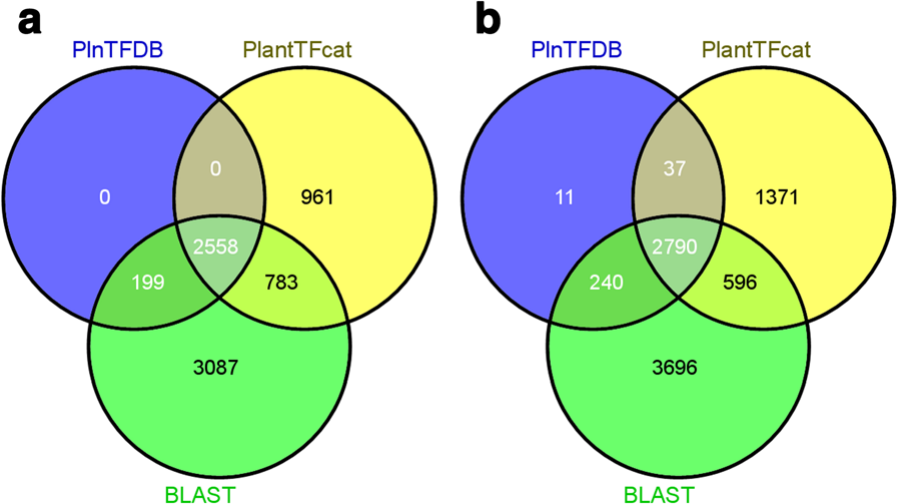
**Venn diagrams that show the differences in prediction results between PlantTFcat and BLAST-based methods.** PlnTFDB represents the TF/TR dataset downloaded from PlnTFDB, PlantTFcat represents the PlantTFcat predictions, and BLAST represents the BLAST search predictions. **(a)** The results using the *Arabidopsis thaliana* gene models release 8 (TAIR8) as the test dataset. **(b)** The results using the *Populus trichocarpa* JGI gene models v1.1 as the test dataset.

We compared the false positive rate of PlantTFcat with traditional BLAST-based methods. The *Arabidopsis thaliana* TAIR8 was chosen as a test dataset. A BLAST search (e-value < =1e-04) was run against the TAIR8 TF/TR genes that were downloaded from PlnTFDB. The BLAST search correctly hit all of the 2,757 TF/TR reference genes because the test dataset contains these reference genes. In addition, the BLAST-based method reported 3,870 more homologous genes as TF/TR candidates that had been excluded by PlnTFDB (Figure 3a). In contrast, PlantTFcat reported only 95 false positives, as described above. We also tested these methods against the *Populus trichocarpa* JGI gene models v1.1 and achieved similar results (Figure 3b). These results suggest that PlantTFcat is a better choice for TF/TR/CR gene annotation over a traditional BLAST search against a reference dataset due to a lower rate of false positives. The details for both comparisons are available in Additional file 4.

## Discussion

The back-end pipeline of PlantTFcat was originally developed to annotate *Medicago truncatula* TFs and other TRs as a part of the International Medicago Genome Sequencing and Annotation Project. The latest genome analysis and gene models from this project were recently published [15]. The PlantTFcat web interface and server were made publicly available in late 2011.

The PlantTFcat web server provides the scientific community with a convenient high-throughput tool to annotate TF/TR/CR genes on a genomic scale. Compared with BLAST-based methods, PlantTFcat does not require biologists to install Linux command line programs, parse outputs, or deploy programs on complicated high performance clusters. With the support of our parallel computing platform and customized InterProScan domain pattern libraries, PlantTFcat is able to analyze large-scale datasets, such as the data from the next generation sequencing platform for the genomic sequencing projects.

PlantTFcat uses signature domain information to ascribe proteins to different families depending on the presence or absence of multiple domains in a single protein, which are based on published TF/TR/CR functional characterizations. Our performance evaluations indicated that PlantTFcat identifies TF/TR/CR genes with a lower false positive rate than traditional BLAST-based approaches without compromising the true positive rate.

PlantTFcat is also able to distinguish families with “*prohibited domain”* patterns. For example, the B3, AP2-EREBP, RAV, and ARF families have common domains, but exclude some domains in their sequences. The B3 and AP2-EREBP families each contain one DNA binding domain, the B3 DNA binding domain and the AP2 DNA binding domain, respectively. The RAV family contains both the B3 and AP2 DNA binding domains in a single protein, and the ARF family contains a B3 DNA binding domain as well as a protein-protein interaction domain. Such complicated families cannot be predicted easily with a single BLAST search. For example, on the NCBI website, At3g25730.1 is annotated as an AP2/ERF gene as well as a B3 family transcription factor, namely ARF14. However, At3g25730.1 is actually a member of the RAV family because it contains both B3 and AP2 domains.

## Conclusions

In conclusion, PlantTFcat performs a systematic analysis of protein domain signatures in InterProScan to produce high coverage and sensitivity for TF/TR/CR annotations. PlantTFcat provides more accurate functional classifications than BLAST-based methods.

## Availability and requirements

**Project name:** PlantTFcat

**Project home page:**http://plantgrn.noble.org/PlantTFcat/

**Operating system:** Platform independent (web-based application)

**Programming language:** Groovy, Java

**Other requirements:** A web browser

**License:** None for usage.

**Any restrictions to use by non-academics:** None.

## Competing interests

The authors declare that they have no competing interests.

## Authors’ contributions

PZ conceived the project and supervised the development of the PlantTFcat system as well as the analyses. XD developed the PlantTFcat system and performed the system performance evaluations. SS and MU provided in-depth insights into chromatin regulators, transcription factors, and transcriptional regulators, curated the mapping table between the InterProScan domains and the TF/TR families, and assisted with the compilation and fine-tuning of the prediction logics. XD, SS, and PZ wrote the manuscript. All authors read and approved the final manuscript.

## Supplementary Material

Additional file 1 List of comprehensive prediction logics for all TF/TR/CR families included in the PlantTFcat. Click here for file

Additional file 2 **Performance analysis by systematically comparing PlantTFcat predictions and the PlnTFDB database on *Arabidopsis thaliana *** **genome.** Click here for file

Additional file 3 **Performance analysis by systematically comparing PlantTFcat predictions and the PlnTFDB database on Maize (*Zea mays*) genome.** Click here for file

Additional file 4 **Performance comparisons between PlantTFcat and traditional BLAST method on *Arabidopsis thaliana *** **genome.** Click here for file
